# Impact of Feed Intake and Body Fat Deposition on Reproductive Performance and Placental Function of Shaziling Sows: A Pilot Study

**DOI:** 10.3390/ani16172785

**Published:** 2026-09-04

**Authors:** Luyao Gao, Huadi Mei, Fuxuan Xiong, Liang Chen, Yulian Li, Hong Tan, Shusong Wu, Jianhua He

**Affiliations:** 1 College of Animal Science and Technology, Hunan Agriculture University, Changsha 410128, China; 18839835979@163.com (L.G.);; 2 State Key Laboratory of Animal Nutrition, Institute of Animal Sciences, Chinese Academy of Agricultural Sciences, Beijing 100081, China; 3 Animal Breeding Station of Xiangtan, Xiangtan 411104, China

**Keywords:** body fat deposition, feed intake regulation, gut microbiota, placental function, reproductive performance, Shaziling sow

## Abstract

Excessive body fat deposition during pregnancy is believed to have an adverse effect on the reproductive performance of sows. Feed intake regulation (REG) is an effective method to modulate energy intake and fat deposition. This study aimed to explore the impact of feed intake and body fat deposition on reproductive performance. A total of 24 Shaziling sows (parity 3–6) were divided into two groups based on their BF on day 30 of gestation, and subsequently randomly assigned to control (CON) and REG groups: (1) BF between 12 and 16 mm (LB-CON, *n* = 6), (2) BF between 12 and 16 mm (LB-IF, *n* = 6), (3) BF between 18 and 22 mm (HB-CON, *n* = 6), and (4) BF between 18 and 22 mm (HB-RF, *n* = 6). Sows in the LB-CON and HB-CON groups were fed a basic diet at a dosage of 1.88 kg/d. The LB-IF and HB-RF groups received REG and were fed a basic diet at dosages of 2.07 kg/d and 1.69 kg/d, respectively. Higher total litter weight and live litter weight were observed in HB-RF sows (*p* < 0.05). In addition, HB-CON sows displayed higher serum levels of IL-6, TG, and GLU when compared with LB-CON sows (*p* < 0.05), whereas HB-RF sows exhibited lower levels of these markers than HB-CON sows (*p* < 0.05). The lipid droplet area fraction was higher in HB-CON sows than in LB-CON sows (*p* < 0.05), and this fraction was reduced in HB-RF sows compared with HB-CON sows (*p* < 0.05). The placental level of TC increased in HB-CON sows when compared with LB-CON sows (*p* < 0.05). The placental levels of T-AOC and GPX increased in HB-RF sows when compared with HB-CON sows (*p* < 0.05). Moreover, the mRNA expression of FABP3 was higher in HB-CON sows when compared with LB-CON sows (*p* < 0.05). The mRNA expression of GLUT3 and PlGF increased in HB-RF sows when compared with HB-CON sows (*p* < 0.05). In addition, HB-RF sows exhibited increased Shannon, Simpson, and ACE indices when compared with HB-CON sows (*p* < 0.05). The characterization of the fecal microbiota revealed that HB-RF sows displayed a lower ratio of Firmicutes/Bacteroidota and an increased relative abundance of Limosilactobacillus when compared with HB-CON sows (*p* < 0.05). Collectively, these observations suggested that reduced feed intake in Shaziling sows with high backfat thickness improved reproductive indices, alleviated placental oxidative stress and inflammatory status, elevated placental nutrient transport and angiogenesis-related gene expression, and reshaped fecal microbiota. These results provide valuable reference for pig production.

## Simple Summary

Excessive body fat deposition during pregnancy is believed to have an adverse effect on the reproductive performance of sows. Feed intake regulation is an effective method to modulate energy intake and fat deposition. This study aimed to explore the impact of feed intake and body fat deposition on reproductive performance, placental function, and gut microbiota in Shaziling sows. Our findings demonstrate that reduced feed intake improved reproductive performance (e.g., total litter weight and live litter weight) in Shaziling sows with high backfat thickness, and these beneficial effects may be attributed to the enhancement of gene expression associated with placental nutrient transport and angiogenesis. These findings serve as a valuable reference for pig production.

## 1. Introduction

The Shaziling pig, an indigenous Chinese breed, is characterized by strong reproductive capacity, superior meat quality, roughage tolerance, and strong resistance to disease. As a fat-type breed, production of Shaziling pig faces several challenges, such as slow growth, excessive fat deposition, low feed conversion ratio, and low lean meat percentage [1]. Backfat thickness (BF) is an important indicator reflecting the body condition of sows, and both excessive and insufficient body fat deposition in sows have adverse impacts on reproductive performance. In commercial lean-type sows, current nutritional recommendations generally target a BF range of 17 to 21 mm at farrowing to optimize reproductive outcomes and lactation performance [2]. Large White sows with a moderate BF (13 to 20 mm) on day 110 of gestation exhibited the highest litter size of alive piglets and the lowest levels of placental oxidative stress and inflammation when compared with that of low-backfat-thickness (LB, 9 to 12 mm) and high-backfat-thickness (HB, 21–25 mm) groups [3]. However, these BF thresholds were established primarily in lean-type commercial breeds. Indigenous fat-type breeds such as the Shaziling pig exhibit fundamentally different patterns of lipid deposition and metabolism. They accumulate greater subcutaneous and intramuscular fat, possess higher lipogenic enzyme activity, and display strong roughage tolerance compared with commercial lean breeds [1]. These breed-specific physiological differences imply that optimal BF ranges and nutritional strategies derived from commercial sows may not be directly applicable to indigenous fat-type breeds.

It has been reported that excessive BF in sows during pregnancy can cause placental lipotoxicity, impair placental vascular development and function, hinder placental nutrient transport, damage fetal growth, and lead to systemic inflammation and oxidative stress [4,5]. When sows maintain a relatively LB, they need to consume their own fat reserves to sustain pregnancy and metabolic processes, and thus cannot provide sufficient energy and nutrition for the full development of the fetuses [6]. Therefore, maintaining appropriate body fat deposition during pregnancy is crucial for improving the reproductive performance of sows.

The imbalance between energy intake and expenditure is the primary cause of obesity. Implementing nutritional regulation strategies, such as feed intake regulation (REG), is an effective method to modulate energy intake and mitigate obesity [7]. Excessive maternal fat deposition exacerbates metabolic syndrome and insulin resistance in late gestation [8], leading to abnormal accumulation of triglycerides in the placenta and ectopic fat deposition, which in turn causes placental development retardation and impairs function [9]. This placental dysfunction will eventually comprehensively impair the reproductive performance of sows, specifically manifested as a reduction in litter size [10], an increase in the incidence of intrauterine growth restriction [4], and a significant decrease in litter weight [11]. A previous study has shown that high body fat deposition impaired the reproductive performance of Shaziling sows, as evidenced by reductions in total litter weight, average weight of total births, total live birth weight, average weight of live births, and placental efficiency [12]. In addition, the gut microbiota undergoes dramatic remodeling during late pregnancy that can affect a sow’s physiological state and metabolic processes [13]. Presently, the optimal BF range of Shaziling sows with different parities remains undefined, and whether BF recommendations derived from commercial lean breeds are appropriate for Shaziling sows is unknown. In addition, whether REG-mediated body fat deposition alteration changes placental function and reproductive performance of Shaziling sows remains unclear. We hypothesized that moderate REG during gestation would regulate body fat deposition in Shaziling sows, thereby improving placental function and reproductive performance. To test this hypothesis, this study aims to explore the impact of feed intake and body fat deposition on reproductive performance, placental function, and gut microbiota of Shaziling sows.

## 2. Materials and Methods

### 2.1. Animal Ethics Statement

All designs and procedures involving animals were conducted following the protocol approved by the Animal Care and Use Committee of Hunan Agricultural University (Approval Number 20240331), and performed according to the guidelines outlined in the Regulations on the Administration of Laboratory Animals.

### 2.2. Animals and Experimental Design

This experiment was conducted at the Shaziling Pig Breeding Farm located in Xiangtan (Xiangtan, China). A total of 24 multiparous (3–6 parity) Shaziling sows were divided into two groups based on their BF on day 30 of gestation, and subsequently randomly assigned to control (CON) and REG groups: (1) BF between 12 and 16 mm (LB-CON, 3.67 ± 0.52 parity), (2) BF between 12 and 16 mm (LB-IF, 3.83 ± 1.17 parity), (3) BF between 18 and 22 mm (HB-CON, 3.17 ± 0.41 parity), and (4) BF between 18 and 22 mm (HB-RF, 3.50 ± 0.84 parity). Sows in each group were fed a basic diet. According to the feeding level advised by a local farm practice, sows in the LB-CON and HB-CON groups were fed a basic diet at a dosage of 1.88 kg/d. The LB-IF and HB-RF groups were subjected to REG and fed a basic diet at dosages of 2.07 kg/d and 1.69 kg/d, respectively. From days 30 to 85 of gestation, corresponding nutritional regulation strategies were implemented, and then normal feed intake (2.3 kg/d) was resumed until delivery. They were fed at 07:30 and 16:00 every day during the experiment, with half of the daily feed given at each meal. The basal diet was formulated to meet the nutrient requirements for gestational sows as recommended by the Nutrient Requirements of Swine in China (China National Standard, GB/T 39235-2020 [14]), and its composition is shown in Supplementary Table S1. Sows had free access to water throughout the experiment and were transferred to individual farrowing crates on day 110 of gestation. The temperature in the farrowing room was kept between 20 and 25 °C using the ventilation system.

### 2.3. Data and Sample Collection

On days 30, 85, and 110 of gestation, the BF was measured at the P2 position (left side of the 10th rib and 6 cm lateral to the spine) using an ultrasound machine (BXLV50, Zhengzhou Boxianglai Electronic Technology Co., Ltd., Zhengzhou, China), which was performed by the same person. The BF was measured three times and the average value was taken. On the day of delivery, reproductive performance data were collected, including the litter size of total births and alive births, total litter weight, alive litter weight, placental weight, and placental efficiency (total litter weight/total placental weight). Within 2 h after parturition, blood samples were collected from sows by the ear vein. For placenta sampling, two placentas (about 10 g of the placenta, 3 to 4 cm from the cord insertion point) were randomly selected from each sow at the time of collection. To ensure the quality and representativeness of the samples, we applied the following exclusion criteria: placentas showing obvious calcification or yellowish discoloration were excluded from placenta sampling. A portion of the placental samples was placed in liquid nitrogen immediately for rapid freezing and stored at −80 °C for further analysis, and the remaining portion was fixed with 4% paraformaldehyde for hematoxylin and eosin (HE) staining and Oil Red O staining. Blood samples were left standing at room temperature for 30 min, centrifuged at 1500× *g* and 4 °C for 10 min, and the supernatants were collected and stored at −80 °C for subsequent analysis. Fresh fecal samples were collected directly into sterile 2 mL cryopreservation tubes by massaging the rectum of sows on day 110 of gestation, and then stored at −80 °C until further analysis.

### 2.4. Serum Biochemical Indexes and Placental Glucose and Lipid Metabolism, Antioxidant, and Inflammation

The serum and placental levels of tumor necrosis factor-α (TNF-α), interleukin (IL)-1β, IL-6, and insulin (INS) were determined by porcine-specific ELISA kits (Baipeng Biotech, Chendu, China, catalog No. BP03201, BP02654, BP01228, BP03793). The serum and placental levels of total cholesterol (TC), triglyceride (TG), total antioxidant capacity (T-AOC), glutathione peroxidase (GPX), and malondialdehyde (MDA) as well as the serum levels of glucose (GLU), high-density lipoprotein (HDL), and low-density lipoprotein (LDL) were measured using commercial assay kits (Beijing Box Biotech, Beijing, China, catalog No. AKAO012M, AKFA013M, AKFA003M, AKFA002M, AKBL011M, AKBL012M, AKSU001M).

### 2.5. Histology

Fixed samples of placenta were dehydrated, paraffin-embedded, sectioned, dewaxed, and stained with H&E according to the procedure described in our previous study [13]. Briefly, placental tissues were fixed in formalin overnight at 4 °C. Following dehydration in 50% ethanol, tissues were embedded in paraffin and sectioned. Sections were sequentially placed in xylene I and xylene II (20 min each), absolute ethanol I and II (5 min each), and 75% ethanol (5 min), and then washed with tap water. The sections were subsequently stained with H&E solution and sealed with neutral balsam. Finally, microscopy and image acquisition were performed using an MShot Biological Microscope ML31 (Guangzhou Mingmei Technology Co., Ltd., Guangzhou, China). Paraffin-embedded placental sections were whole-slide scanned using CaseViewer (version 2.4). For each sow, six randomly selected non-overlapping microscopic fields (100× magnification, 10× objective) were obtained. Regions containing large blood vessels, tissue folds, and sectioning artifacts were excluded from analysis. ImageJ (v1.54f) was used for vessel counting and valid area measurement. Blood vessels with intact endothelium and clear lumens were counted, and vascular density was calculated subsequently.

Oil Red O staining was performed on placental frozen sections according to our previous study [13]. Briefly, the oil red O working solution was prepared by mixing saturated oil red O stock solution with distilled water at a 6:4 ratio, incubating overnight at 4 °C, filtering through qualitative filter paper, storing at 4 °C for 24 h, and performing a second filtration. Sections were stained with oil red O working solution for 8 to 10 min in the dark. They were then removed and allowed to stand for 3 s before being differentiated in two cylinders of 60% isopropanol (3 s and 5 s, respectively). After rinsing in two cylinders of distilled water (10 s each) and draining for 3 s, sections were counterstained with hematoxylin for 3 to 5 min, immersed sequentially in three cylinders of distilled water (5 s, 10 s, and 30 s), differentiated for 2 to 8 s, washed in two cylinders of distilled water (10 s each), and placed in blue solution for 1 s. Following immersion in two cylinders of tap water (5 s and 10 s, respectively), the staining effect was observed microscopically. Sections were then sealed with glycerol gelatin and subjected to microscopic examination, image acquisition, and analysis. Oil Red O-stained placental frozen sections were whole-slide scanned using SaiViewer (version 3.0.1), and images were exported at 40× magnification (4× objective) for lipid droplet quantification. Six non-overlapping random microscopic fields were selected per sow, and six sows were included in each group. Quantitative analysis was performed using ImageJ software.

### 2.6. Real-Time Quantitative PCR (RT-qPCR)

Total RNA isolation, cDNA synthesis, and RT-qPCR were performed according to our previous study [12]. Total RNA from placental samples was extracted using total RNA Extraction Kits (Steady Pure Universal RNA Extraction Kit, Accurate Biotechnology, AG21017, Changsha, China). The purity and concentration of the obtained total RNA were determined with a NanoDrop-1000 spectrophotometer (Thermo Fisher Scientific Inc., Waltham, MA, USA). Subsequently, the cDNA was synthesized using a Reverse Transcription Kit (Evo M-MLV RT Mix Kit with gDNA Clean for qPCR Ver.2, Accurate Biotechnology, AG11728). RT-qPCR of the target genes and housekeeping gene β-actin was performed on a CFX Connect detection system (Bio-Rad Laboratories, Hercules, CA, USA) using the QPCR Premix Kit (SYBR Green Premix Pro Taq HS qPCR Kit, Accurate Biotechnology, AG11701) in a total volume of 10 μL and analyzed in triplicate. Primers used for selected genes are presented in Supplementary Table S2. The protocols for all genes included a denaturation program (95 °C for 3 min), an amplification and quantification program repeated for 40 cycles (95 °C for 5 s, 60 °C for 30 s), followed by the melting curve program at 65–95 °C with a heating rate of 0.1 °C/s. The relative quantification of gene amplification by Real-Time PCR was determined using the value of the threshold cycle (Ct). The housekeeping gene β-actin was used to normalize each target gene expression level, and the relative expression levels of the target genes were quantified by the 2^−∆∆Ct^ method.

### 2.7. 16S rRNA Sequencing

The fecal microbiota was characterized by 16S rDNA gene sequencing as described previously [15]. Fecal samples from Shaziling sows were collected. Genomic DNA was extracted using a commercial kit (MoBio Laboratories, Carlsbad, CA, USA). The V3–V4 hypervariable region of the 16S rRNA gene was amplified by PCR with forward primer 341F (5′-CCTAYGGGRBGCASCAG-3′) and reverse primer 806R (5′-GGACTACHVGGGTWTCTAAT-3′). The PCR cycling parameters were set as follows: an initial denaturation at 94 °C for 2 min; 30 cycles consisting of 94 °C for 30 s, 55 °C for 30 s, and 72 °C for 30 s; and a final extension at 72 °C for 10 min. Subsequently, PCR amplicons were purified using the AxyPrep DNA Gel Extraction Kit (Axygen Biosciences, Union City, CA, USA), and the purified products were quantified on a Quantus™ Fluorometer (Promega, Madison, WI, USA). Qualified sequencing libraries were constructed and sequenced on the MGI G99 platform. All raw 16S rRNA amplicon sequencing reads of sow fecal samples were deposited in the NCBI Sequence Read Archive (SRA) under BioProject accession PRJNA1509686. Raw sequencing data will be publicly released upon manuscript publication. Bioinformatics analysis was performed using the online platform provided by Metware Biotechnology Co., Ltd. (Wuhan, China).

### 2.8. Statistical Analysis

All data were expressed as mean ± SEM. An analysis of similarity (ANOSIM) was used to reveal whether the β diversity was significantly different across groups based on the weighted UniFrac matrices using the ‘vegan’ package in the R program. The effect of REG on BF was analyzed using a linear mixed model. For other data, their normality and homogeneity of variance were verified, and one-way ANOVA was performed with GraphPad Prism 8.0.0 (GraphPad Software, San Diego, CA, USA). If the data did not conform to the assumption of normality and homogeneity of variances, a generalized linear model was applied. The appropriate generalized linear model was selected based on the data type of each response variable, i.e., count-derived variables versus continuous variables. All data were evaluated using the sow as the experimental unit. Tukey’s multiple-comparisons test was used to evaluate the difference among the treatments. Differences were considered significant at *p* < 0.05.

## 3. Results

### 3.1. Effects of REG on BF and Reproductive Performance in Shaziling Sows

**Figure 1:**
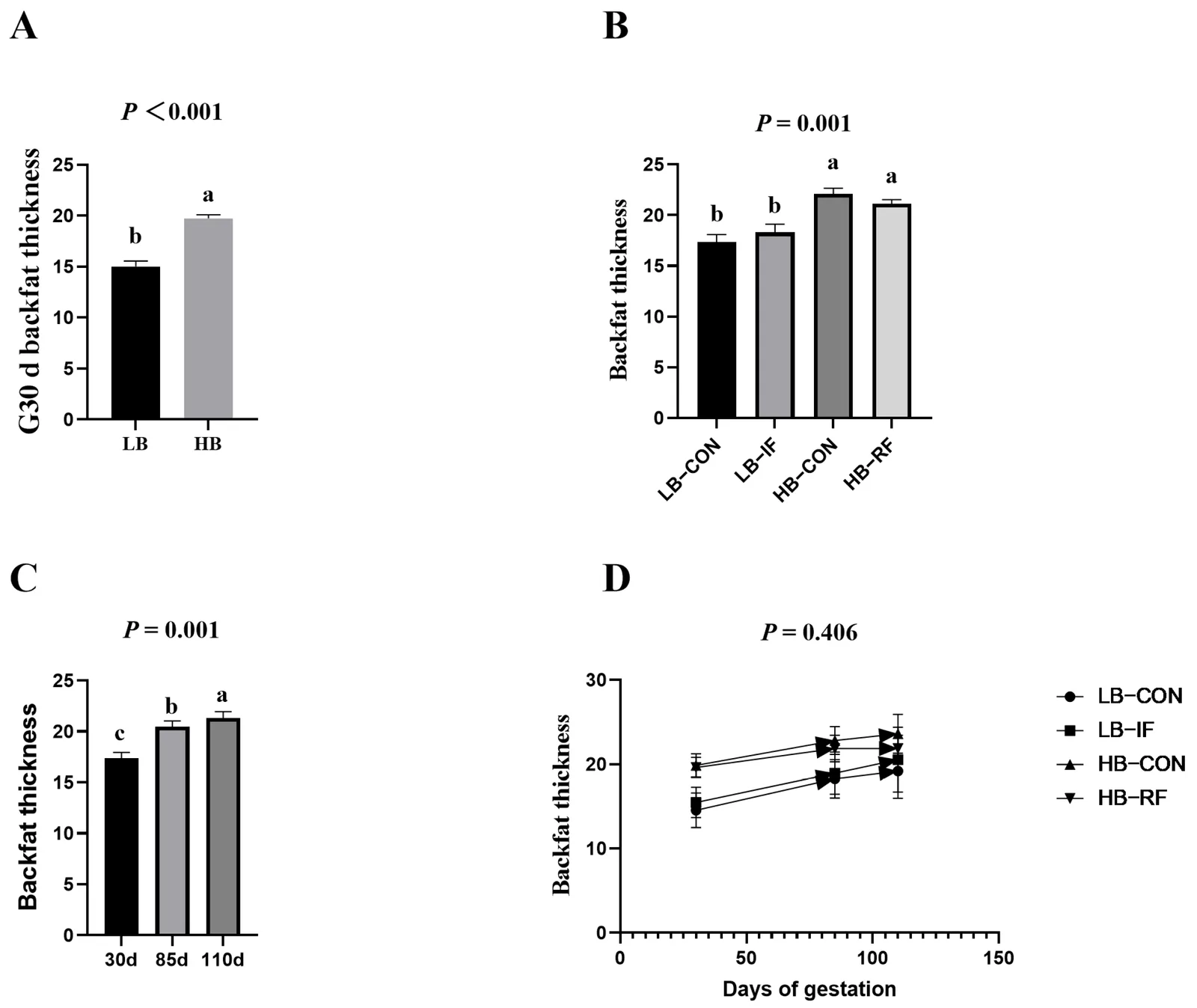
The effect of REG on BF in Shaziling sows. (**A**) BF at 30 days of gestation (*n* = 12). (**B**) BF in different groups. (**C**) BF at different gestation stages. (**D**) BF at 30, 85, and 110 days of gestation. LB, backfat thickness of sows ranges from 12 to 16 mm. HB, backfat thickness of sows ranges from 18 to 22 mm. Sows in the LB-CON and HB-CON groups were fed a basic diet at a dosage of 1.88 kg/d. The LB-IF and HB-RF groups were fed a basic diet at dosages of 2.07 kg/d and 1.69 kg/d, respectively. Values are presented as the means ± SEM (*n* = 6). The different superscript letters on the same row indicate significant differences (*p* < 0.05).

As shown in Figure 1A, BF in HB sows was significantly higher than that in LB sows on day 30 of gestation (*p* < 0.05). As shown in Figure 1B, BF in HB-CON sows was significantly higher than that in LB-CON sows (*p* < 0.05), but neither increased feed intake in LB sows nor decreased feed intake in HB sows had a significant effect on BF (*p* > 0.05). In addition, the gestation stage had a marked influence on BF, with values increasing as gestation progressed and reaching a maximum on day 110 of gestation (*p* < 0.05).

**Figure 2:**
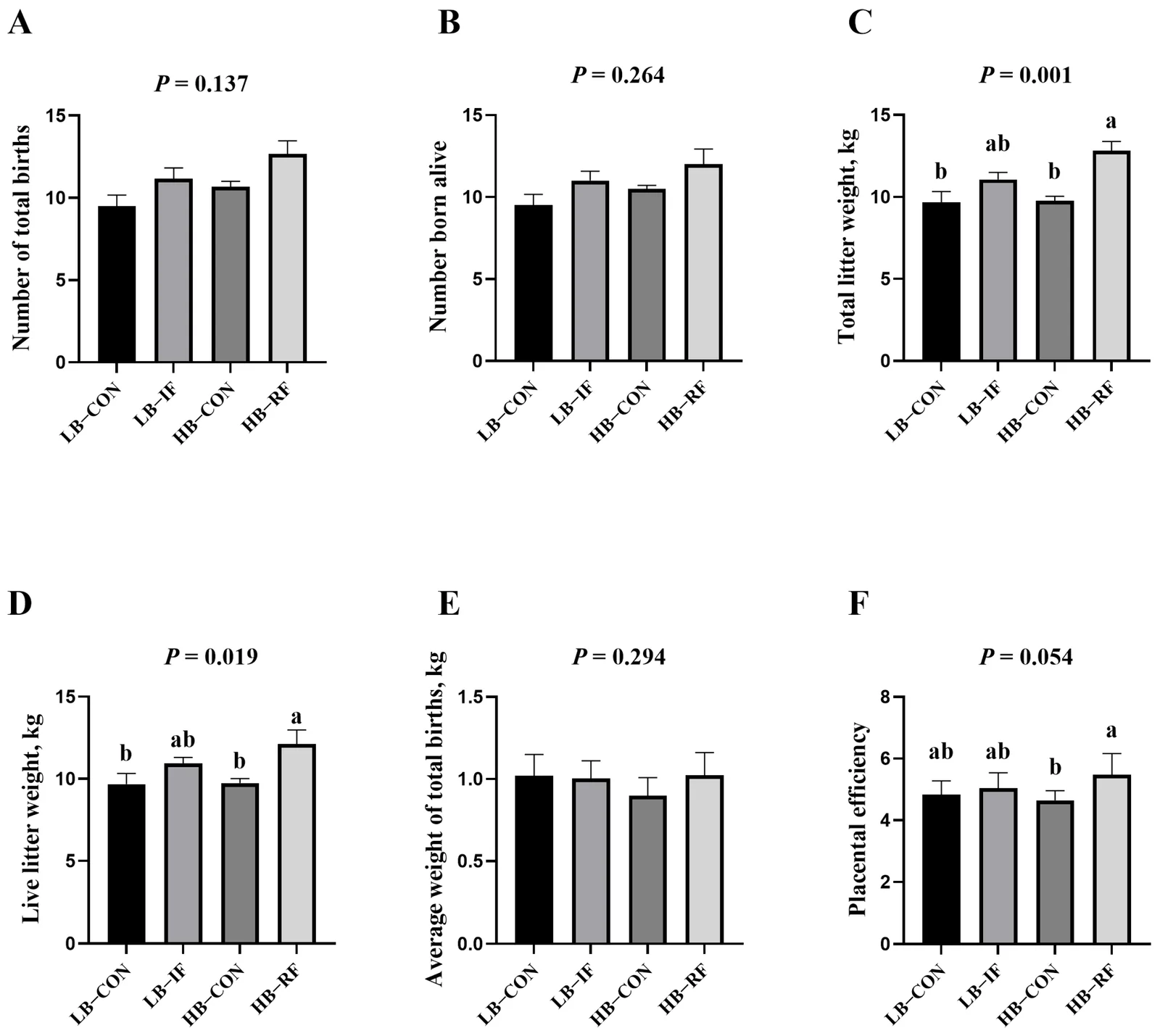
Effects of REG on reproductive performance and placental efficiency in Shaziling sows. (**A**) litter size of total births, (**B**) litter size of alive births, (**C**) total litter weight, (**D**) alive litter weight, (**E**) average weight of total births, (**F**) placental efficiency. Values are presented as the means ± SEM (*n* = 6). The different superscript letters on the same row indicate significant differences (*p* < 0.05).

As shown in Figure 2, increased total litter weight and alive litter weight were observed in HB-RF sows when compared with HB-CON sows (*p* < 0.05).

### 3.2. Effects of REG on Serum Biochemistry and Inflammatory Factors in Shaziling Sows

**Figure 3:**
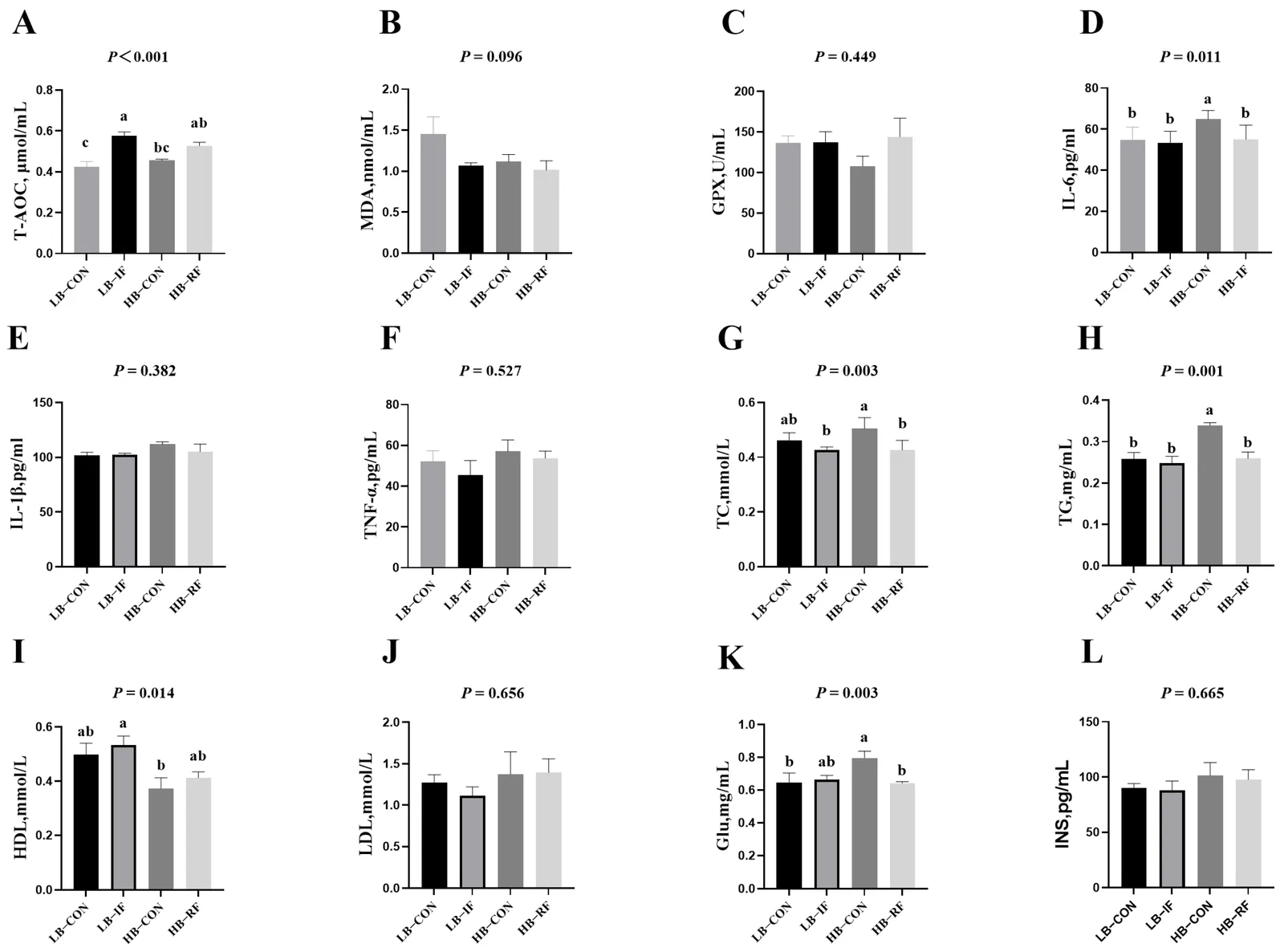
Effects of REG on serum biochemistry and inflammatory factors in Shaziling sows. The serum levels of (**A**) total antioxidant capacity (T-AOC), (**B**) malondialdehyde (MDA), (**C**) glutathione peroxidase (GPX), (**D**) interleukin-6 (IL-6), (**E**) interleukin-1β (IL-1β), (**F**) tumor necrosis factor-α (TNF-α), (**G**) total cholesterol (TC), (**H**) triglyceride (TG), (**I**) high-density lipoprotein (HDL), (**J**) low-density lipoprotein (LDL), (**K**) glucose (GLU), (**L**) insulin (INS). Values are presented as the means ± SEM (*n* = 6). The different superscript letters on the same row indicate significant differences (*p* < 0.05).

As shown in Figure 3A–L, serum levels of IL-6, TG, and GLU were higher in HB-CON sows than in LB-CON sows (*p* < 0.05), whereas HB-RF sows exhibited lower levels of these markers than HB-CON sows (*p* < 0.05). In addition, an increased serum T-AOC level was observed in LB-IF sows when compared with LB-CON sows (*p* < 0.05).

### 3.3. Effects of REG on Placental Fat Deposition, Oxidation, and Inflammatory Status in Shaziling Sows

The Oil Red O staining of the placenta of Shaziling sows is shown in Figure S1B,D. The lipid droplets in the HB-CON group exhibited deeper staining intensity and were more numerous in quantity when compared with the LB-CON group. Further quantitative results indicated that the lipid droplet area fraction was higher in the HB-CON group than in the LB-CON group (*p* < 0.05), and that this fraction was reduced in the HB-RF group compared with the HB-CON group (*p* < 0.05).

**Figure 4:**
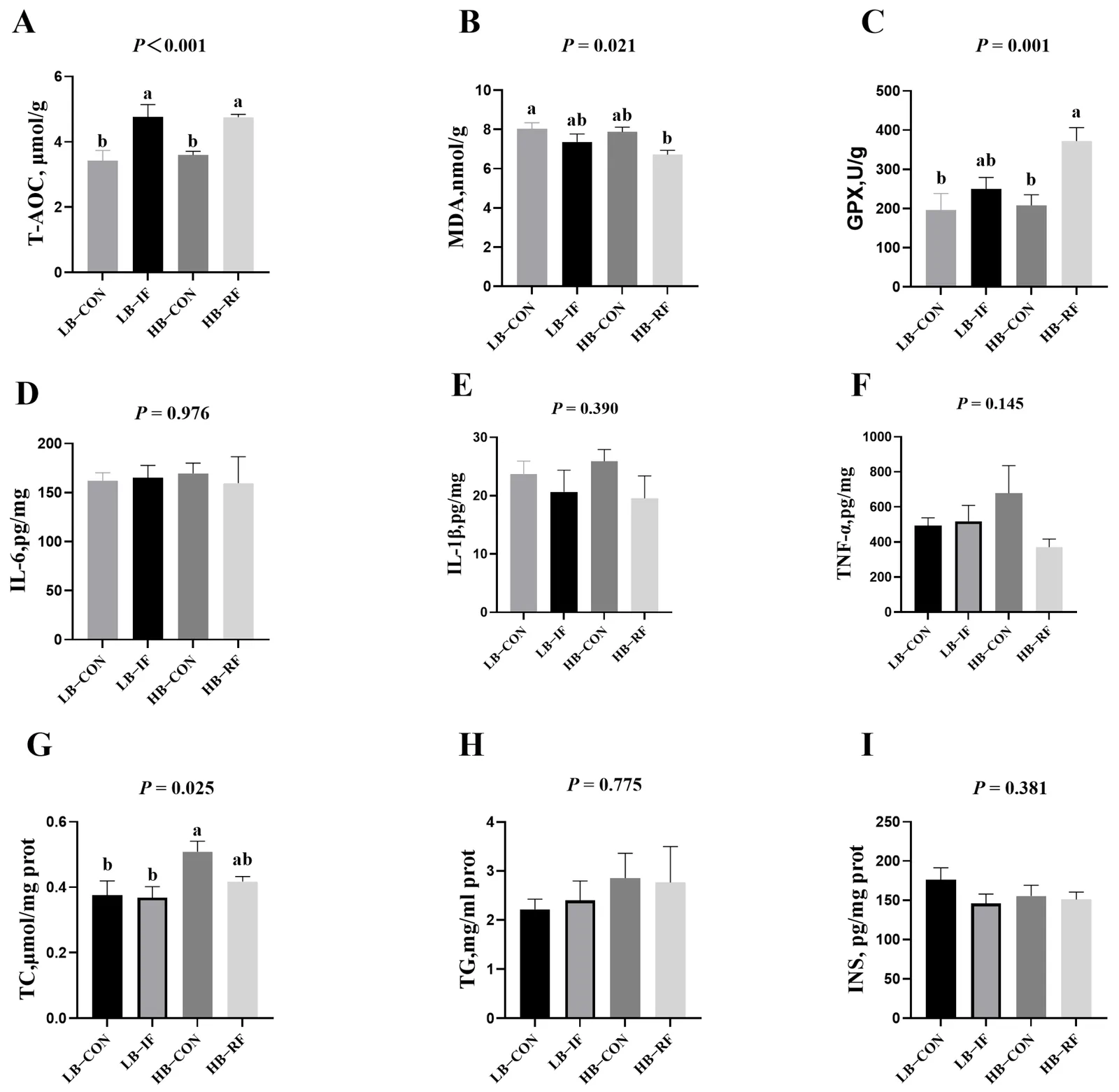
Effects of REG on placental oxidation and inflammatory status in Shaziling sows. The placental levels of (**A**) (T-AOC), (**B**) malondialdehyde (MDA), (**C**) glutathione peroxidase (GPX), (**D**) interleukin-6 (IL-6), (**E**) interleukin-1β (IL-1β), (**F**) tumor necrosis factor-α (TNF-α), (**G**) total cholesterol (TC), (**H**) triglyceride (TG), (**I**) insulin (INS). Values are presented as the means ± SEM (*n* = 6). The different superscript letters on the same row indicate significant differences (*p* < 0.05).

Figure 4A–I showed that the level of TC increased in HB-CON sows when compared with LB-CON sows (*p* < 0.05). The levels of T-AOC and GPX increased in HB-RF sows when compared with HB-CON sows (*p* < 0.05). Moreover, the level of T-AOC increased in LB-IF sows when compared with LB-CON sows (*p* < 0.05). Further detection of placental genes associated with antioxidant capacity and inflammation (Figure S2A–D) showed that TNF-α mRNA expression increased in HB-CON sows when compared with LB-CON sows (*p* < 0.05). In addition, GPX-4 mRNA expression increased in LB-IF sows when compared with LB-CON sows (*p* < 0.05).

### 3.4. Effects of REG on Placental Genes Related to Angiogenesis and Nutrient Transport in Shaziling Sows

**Figure 5:**
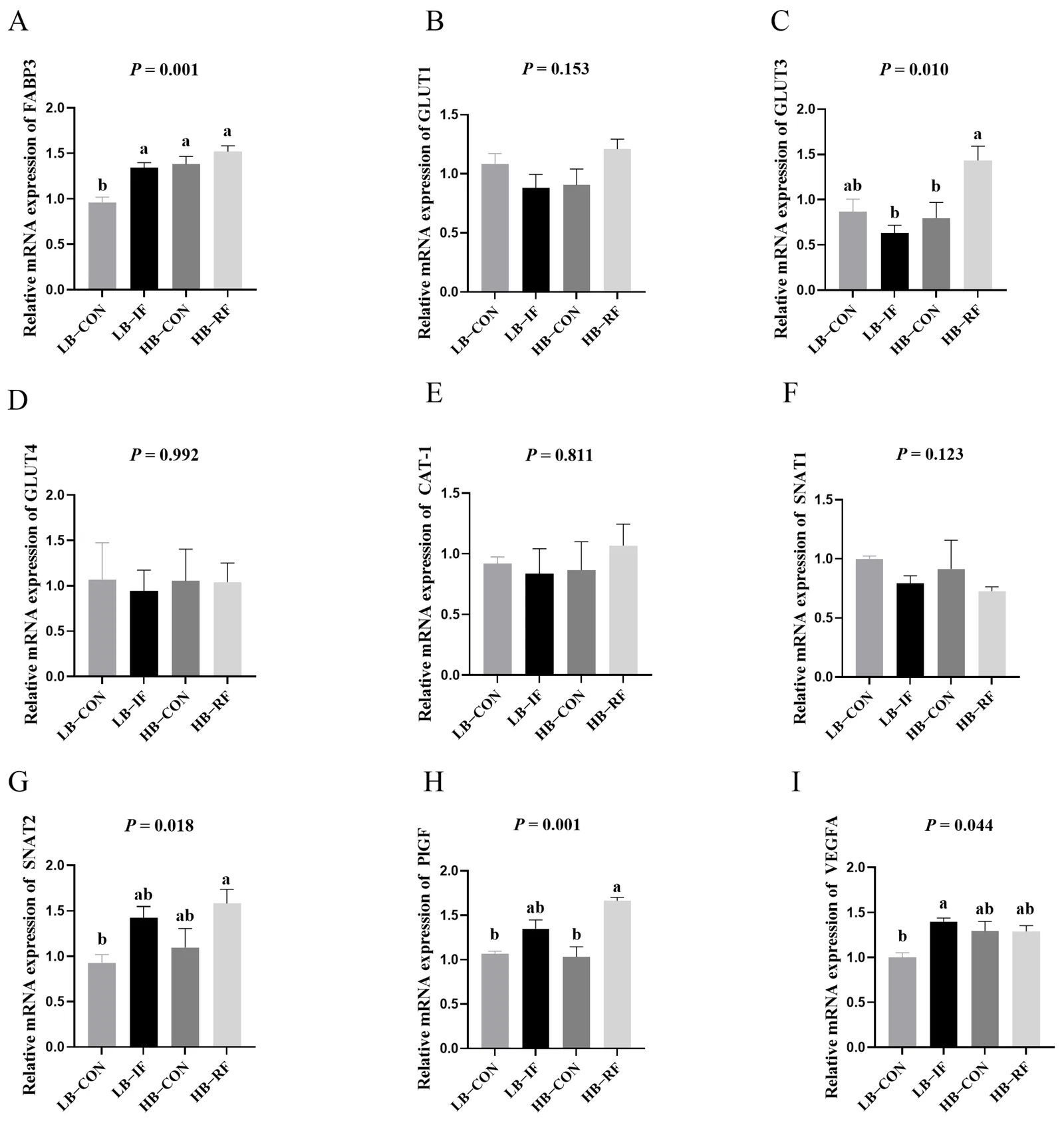
Effects of REG on placental genes related to angiogenesis and nutrient transport in Shaziling sows. The relative mRNA expression levels of (**A**) fatty acid-binding protein 3 (FABP3), (**B**) glucose transporter 1 (GLUT1), (**C**) glucose transporter 3 (GLUT3), (**D**) glucose transporter 4 (GLUT4), (**E**) solute carrier family 7 member 1 (CAT-1), (**F**) sodium coupled neutral amino acid transporter 1 (SNAT1), (**G**) sodium coupled neutral amino acid transporter 2 (SNAT2), (**H**) placental growth factor (PlGF), (**I**) vascular endothelial growth factor A (VEGFA). Values are presented as the means ± SEM (*n* = 6). The different superscript letters on the same row indicate significant differences (*p* < 0.05).

As shown in Figure 5A–I, the mRNA expression of FABP3 was higher in HB-CON sows when compared with LB-CON sows (*p* < 0.05). The mRNA expression of GLUT3 and PlGF increased in HB-RF sows when compared with HB-CON sows (*p* < 0.05). In addition, LB-IF sows exhibited increased mRNA expression of FABP3 and VEGFA when compared with LB-CON sows (*p* < 0.05).

### 3.5. Effects of REG on the Fecal Microbial Community in Shaziling Sows

**Figure 6:**
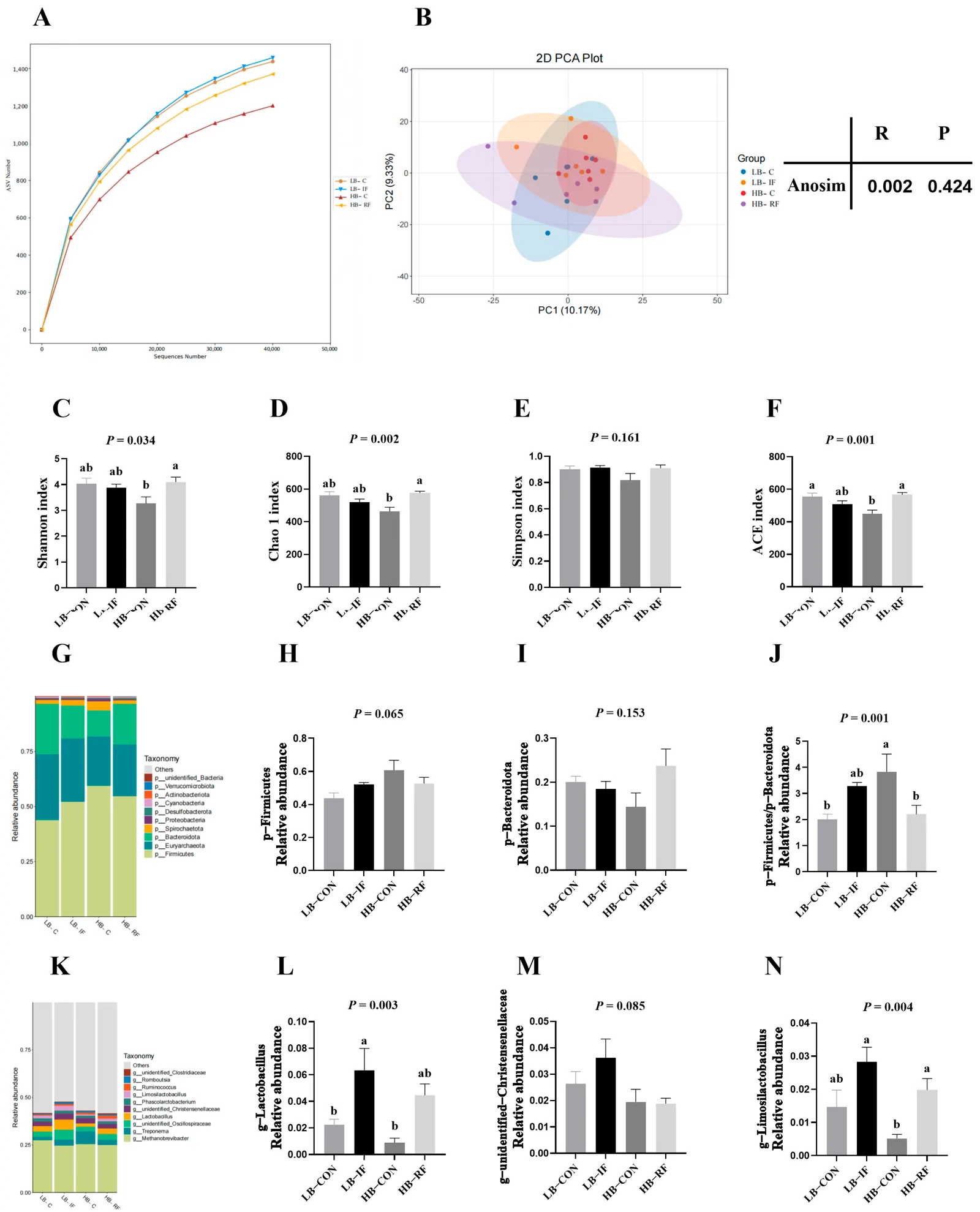
Effects of REG on the fecal microbial community in Shaziling sows. (**A**) Rarefaction curves of bacterial communities for all samples. (**B**) Principal component analysis (PCA) of fecal flora. (**C**–**F**) Alpha diversity of fecal flora. (**G**–**N**) The relative abundance of fecal microbiota composition at the phylum and genus levels. Values are presented as the means ± SEM (*n* = 6). The different superscript letters on the same row indicate significant differences (*p* < 0.05).

A total of 1,959,973 effective tags were obtained from all experimental samples (*n* = 24), ranging from 71,082 to 106,438 effective tags per sample, with an average of 81,666. Good’s coverage for all samples was higher than 99%. The rarefaction curves tended to plateau (Figure 6A), indicating that the sequencing depth was sufficient to characterize the bacterial communities for subsequent α and β diversity analyses. The PCA map showed no separation among the four groups of flora (Figure 6B). ANOSIM confirmed no significant separation of fecal flora among the groups (*p* > 0.05) (Figure 6B).

Figure 6C–F indicated that HB-CON sows displayed a decreased ACE index when compared with LB-CON sows (*p* < 0.05), and HB-RF sows exhibited increased Shannon, Simpson, and ACE indices when compared with HB-CON sows (*p* < 0.05). At the phylum level, the fecal flora consisted mainly of Firmicutes, Euryarchaeota, Bacteroidota, Spirochaetota, Proteobacteria, Desulfobacterota, Cyanobacteria, Actinobacteriota, and Verrucomicrobiota in sows (Figure 6G). Firmicutes, Euryarchaeota, and Bacteroidota were the dominant phyla. The HB-CON sows displayed a higher ratio of Firmicutes/Bacteroidota when compared with LB-CON sows (*p* < 0.05), and this ratio was decreased in HB-RF sows when compared with HB-CON sows (*p* < 0.05). At the genus level (top 10), the fecal flora consisted mainly of *Methanobrevibacter*, *Treponema*, unidentified_Oscillospiraceae, *Lactobacillus*, unidentified_Christensenellaceae, *Phascolarctobacterium*, *Limosilactobacillus*, *Ruminococcus*, *Romboutsia*, and unidentified_Clostridiaceae (Figure 6K). The relative abundance of *Limosilactobacillus* increased in HB-RF sows when compared with HB-CON sows (*p* < 0.05). Moreover, the relative abundance of *Lactobacillus* increased in LB-IF sows when compared with LB-CON sows (*p* < 0.05).

## 4. Discussion

BF is an important indicator reflecting body condition and is closely associated with body fat deposition. Maintaining an appropriate body condition during gestation is an important contributor to improved reproductive performance. In a previous study, excessive body fat deposition in late gestation was associated with impaired reproductive performance, including reductions in total litter weight, average weight of total births, total live birth weight, average weight of live births, and placental efficiency [12]. However, there were no differences in reproductive performance between HB-CON sows and LB-CON sows. The discrepancy between our findings and previous reports may arise from differences in parity condition and backfat thickness selection criteria. In this study, higher total litter weight and live litter weight were observed in HB-RF sows. Excessive fat deposition during pregnancy can lead to placental lipid toxicity, chronic inflammation, and oxidative stress, thereby causing damage to the development of placental blood vessels and an increase in weak piglets [16,17]. In our study, the placental levels of T-AOC and GPX increased in HB-RF sows when compared with HB-CON sows. This may partly be linked to the improved placental function observed in HB-RF sows under a feed-restricted condition.

Maternal excessive body fat deposition is closely related to chronic low-grade inflammation and imbalance of oxidative stress. A previous study showed that an increase in the BF of sows may be accompanied by an elevated level of MDA in the placenta as well as elevations in the mRNA levels of TNF-α, IL-1β, and IL-6 [5]. Yang et al. [12] found that the serum levels of TNF-α, IL-1β, IL-6, and MDA were increased in HB sows when compared to LB sows. Similar to the results described above, the serum level of IL-6 and the mRNA expression of TNF-α increased in HB-CON sows when compared with LB-CON sows. Notably, the serum level of IL-6 decreased, and placental T-AOC and GPX increased in HB-RF sows when compared with HB-CON sows. Feed intake restriction has been reported to improve the inflammatory state and redox balance by reducing the metabolic intermediate products from the metabolism process of GLU and fatty acids [18,19]. In a study conducted in Bama pigs, a high-fat diet increased the serum levels of TNF-α and IL-6, while these were decreased after weight loss [20]. These results suggested that feed intake restriction in HB-RF sows was associated with alleviated oxidative stress and inflammation, which may contribute to better placental vascular development and transport function.

The HB sows exhibit abnormal lipid metabolism, such as disordered lipid metabolism indicators in serum [5]. The excessive fat deposition and disordered lipid metabolism lead to ectopic fat deposition. This study observed higher serum levels of GLU and TG in HB sows, which may be associated with increased ectopic fat deposition in the placenta. This result is consistent with our previous results [12]. However, lower circulating GLU and TG concentrations, as well as reduced placental ectopic fat deposition, were observed in HB-RF sows. A previous study showed that there was a negative correlation between the placental lipid level and piglet birth weight [4]. The expression of placental nutrient-specific transporters is highly associated with fetal weight [21]. These observations suggested that increased placental ectopic fat deposition may be related to impaired expression of placental nutrient-transport genes. Notably, HB-RF sows displayed higher expression of nutrient transport-related genes (e.g., GLUT3) in their placenta. These results suggested that alleviation of abnormal lipid metabolism in HB-RF sows may be related to reduced ectopic fat deposition and subsequent enhanced expression of nutrient transport-related genes.

Adequate placental angiogenesis is a necessary prerequisite for maintaining normal pregnancy and ensuring fetal development. Low birth weight of piglets is associated with decreased density of vessels in the placenta [22]. The formation of new blood vessels in the placenta depends on the processes of vasculogenesis and angiogenesis [23]. These processes are regulated by VEGFA, PlGF, or their related signaling pathways [24,25]. VEGFA and PlGF can activate downstream angiogenic signals by inducing receptor phosphorylation [26]. This study found that PlGF mRNA expression was higher in HB-RF sows, and this elevated expression may contribute to enhanced placental angiogenesis. This observation may be related to reduced placental ectopic fat deposition.

Accumulating evidence indicates that the gut microbiota exerts profound effects on pregnancy, with the maternal gut microbiome undergoing substantial remodeling across gestation. Microbial richness and diversity rise progressively with advancing pregnancy, and these shifts are tightly linked to host metabolic and reproductive physiology [27]. In this study, a lower Firmicutes/Bacteroidota ratio was observed in HB-RF sows. The ratio of Firmicutes/Bacteroidota is a pivotal index for assessing gut microbiota health. An increase in the ratio of Firmicutes/Bacteroidota has been implicated in obesity and metabolic-related conditions [28]. In addition, increased feed intake enriched Lactobacillus in LB-IF sows, while decreased feed intake enriched Limosilactobacillus in HB-RF sows. According to previous reports, *Lactobacillus* indirectly modulates intestinal immune responses by producing lactic acid and competitively inhibits the proliferation of pathogenic bacteria by secreting antibacterial factors [29,30]. *Lactobacillus* was also reported to be involved in the mitigation of endometritis [31]. Notably, *Lactobacillus* was found to be enriched in sows with high reproductive performance [32]. *Limosilactobacillus*-derived metabolic products have been reported to increase litter size in stress-induced reproductive dysfunction in female mice by modulating the hypothalamic–pituitary–adrenal axis, reducing oxidative stress, and restoring normal estrous cyclicity [33]. Taken together with previous studies, elevations in *Lactobacillus* and *Limosilactobacillus* abundances may be associated with improved reproductive-related health status.

## 5. Conclusions

This pilot study observed that reduced feed intake improved reproductive performance (e.g., total litter weight and live litter weight) in Shaziling sows with high backfat thickness. Also, the alleviation of oxidative stress and inflammation, improvement of lipid metabolism, enhancement of gene expression associated with placental nutrient transport and angiogenesis, and optimization of gut microbiota were observed in the HB-RF group. However, these findings should be interpreted in light of several study limitations, including a small sample size, a single-breed design, the lack of longitudinal metabolic measurements, the absence of fetal tissue analyses, the lack of protein-level validation, the observational nature of microbiota associations, the absence of observer blinding for placental histological quantification, and missing standard-curve-based primer efficiency assessment as well as melting-curve validation for qPCR assays. Additionally, blood sampling was performed within 2 h after parturition, which may be confounded by farrowing-related stress and acute peripartum physiological changes. In addition, microbiota profiling relied solely on 16S rRNA amplicon sequencing without metagenomic, metabolomic, or SCFA-related functional validation. Further validation in larger and more diverse cohorts is warranted. Notably, this study was an exploratory pilot study with a limited sample size. While Tukey’s multiple-comparisons test was applied to control for type I error within each individual endpoint across treatment groups, this approach does not account for the cumulative false-positive risk arising from the simultaneous testing of numerous independent biological endpoints. Consequently, the statistically significant findings reported herein should be interpreted as exploratory and hypothesis-generating conclusions rather than definitive confirmatory conclusions. Future studies with larger sample sizes, predefined primary outcomes, and appropriate multiplicity correction strategies are warranted to validate these preliminary observations.

## Data Availability

All data used in this study are available from the corresponding authors upon reasonable request.

## Supplementary Materials

The following supporting information can be downloaded at: https://www.mdpi.com/article/10.3390/ani16172785/s1, Table S1: Composition and nutrient levels of diets (%, DM basis). Table S2: Primers used for Real-time PCR. Figure S1: Effects of REG on placental fat deposition and vessel density. Figure S2: Effects of REG on placental genes associated with antioxidant and inflammation in Shaziling sows.
