# Supplementary material for: Impact of Feed Intake and Body Fat Deposition on Reproductive Performance and Placental Function of Shaziling Sows: A Pilot Study
Source: Animals (Basel). 2026 Sep 4;16(17):2785. doi: 10.3390/ani16172785 (PMC13564728; doi:10.3390/ani16172785)
Supplement: Supplementary file 1 [file animals-16-02785-s001.zip › animals-4444960-supplementary.pdf]

Supplementary Table S1. Composition and nutrient levels of diets (%, DM basis).

Items	Content
Ingredients	
Corn	41.90
Soybean meal	5.30
Wheat flour	20
Rice bran meal	5.80
Wheat bran	17
Beet mea	4.00
Soybean husk	2.50
Limestone	1.30
Rice husk powder	0.22
Propionic acid (50%)	0.12
Phytase	0.05
$NaHCO_3$	0.30
NaCl	0.40
L-lys HCl (70%)	0.47
L-threonine	0.12
Choline chloride	0.15
DL-Met	0.07
Premix[1)]	0.30
Total	100
Nutrient level[2)]	
NE, MJ/kg	9.20
Crude protein	13.50
Crude fiber	6.30
Ash	5.15
Calcium	0.65
Total phosphorus	0.55

SID Lys	0.65
SID Met	0.24
SID Thr	0.44
SID Trp	0.12
SID Val	0.45

DM = dry matter; NE = net energy; SID = standardized ileal digestibility

1）The premix provided the following per kg of diets：VA 9920 IU,VB_1 2.2 mg，VB_2 10 mg，VB_3 44 mg，VB_5 33 mg，VB_6 3.3 mg，VB_7 220 mg，VB_9 1325 mg，VB_{12} 37 mg，VD_3 1985 IU，VE 66 IU，VK 4.4 mg，Cu 8 mg，Fe 80 mg，Mn 45 mg，Zn 80 mg，I 0.5 mg，Se 0.3 mg。

2）NE and amino acids was a calculated value, while the others were measured values.

Supplementary Table S2. Primers used for Real-time PCR

Name	GenBank number	Sequence	Size, bp
β-actin	XM_021086047.1	F:TCTACACCGCTACCAGTTCG	177
		R:GAGTCCTTCTGGCCCATGC	
VEGFA	NM_214084.1	F:GGCAGAAGGAGACCAGAAACC	144
		R:ACACAGGACGGCTTGAAGATG	
PPAR*γ*	NM_214379.1	F:TCGGGGTTCCACTATGGAGT	196
		R:GCCCAAACCTGATGGCGTTA	
LPL	NM_214286.1	F:GATCAGAGTGAAGGCAGGAGA	155
		R:GTACATTTGCTCAGTTTCAGCCA	
CAT-1	NM_001012613.1	F:GGAACACCTCCGGTCATCTC	130
		R:CCCACGAAGGCATAGAAGCA	
GLUT1	XM_021096908.1	F:GCCTGTTTGTGAACCGCTTT	97
		R:CCCAGTTTGGAGAAGCCCAT	
GLUT3	XM_021092392.1	F:TTCAGAGCACCCAACTACCG	167
		R:GTATTAACCACGCCAGCCCC	
GLUT4	NM_001128433.1	F:CGGTTCCTCATTGGTGCCTA	108
		R:CTGGTTGAGTGTCCCCAAGG	
FABP3	NM_001099931.1	F:TGACAGGAAGGTCAAGTCCAT	158
		R:CGTAAGTGCGAGTGCAAACT	
PlGF	XM_003125167.6	F:AGAGACTGCTGTATGCCCATC	115
		R:CACAACCATGTCAAGTGCGT	
IL-1*β*	NM_214055.1	F:AGACCACAAATCTCTAGTGCTGG	139
		R:GGTCACAGGTATCTTGTTGTTGC	
IL-6	NM_214399.1	F:TCCGGACAAAACTGAAGAACTCA	107
		R:GTACCTCCTTGCTGTTTTCACAC	
TNF-*α*	NM_001159607.1	F:AGAAGCGAAAGATGGGATGCT	109
		R:ACTCATTTGGGAAGCCTGGAG	

GPX4	NM_214407.1	F:AAGCCAACGCTTCTCTGC	175
		R:CATTGGTGACGATGCACACG	
SNAT1	XM_003355629.4	F:AATCACAAGCCAGGATGGAG	180
		R:GCGGTAATTTGACTGTTTCCTTTT	
SNAT2	XM_013997964.2	F:TGATTCTTGCCGACCACGTT	140
		R:TCATTCTTCTACGGCTGCGG	

Abbreviations: CAT-1, solute carrier family 7 member 1; FABP3, fatty acid-binding protein 3; GLUT1, glucose transporter 1; GLUT3, glucose transporter 3; GLUT4, glucose transporter 4; GPX4, glutathione peroxidase; IL-1β, interleukin-1β; IL-6, interleukin-6; LPL, lipoprotein lipase; PlGF, placental growth factor; PPARγ, peroxisome proliferator-activated receptor γ; SNAT1, sodium coupled neutral amino acid transporter 1; SNAT2, sodium coupled neutral amino acid transporter 2; TNF-α tumor necrosis factor-α; VEGFA, vascular endothelial growth factor A.

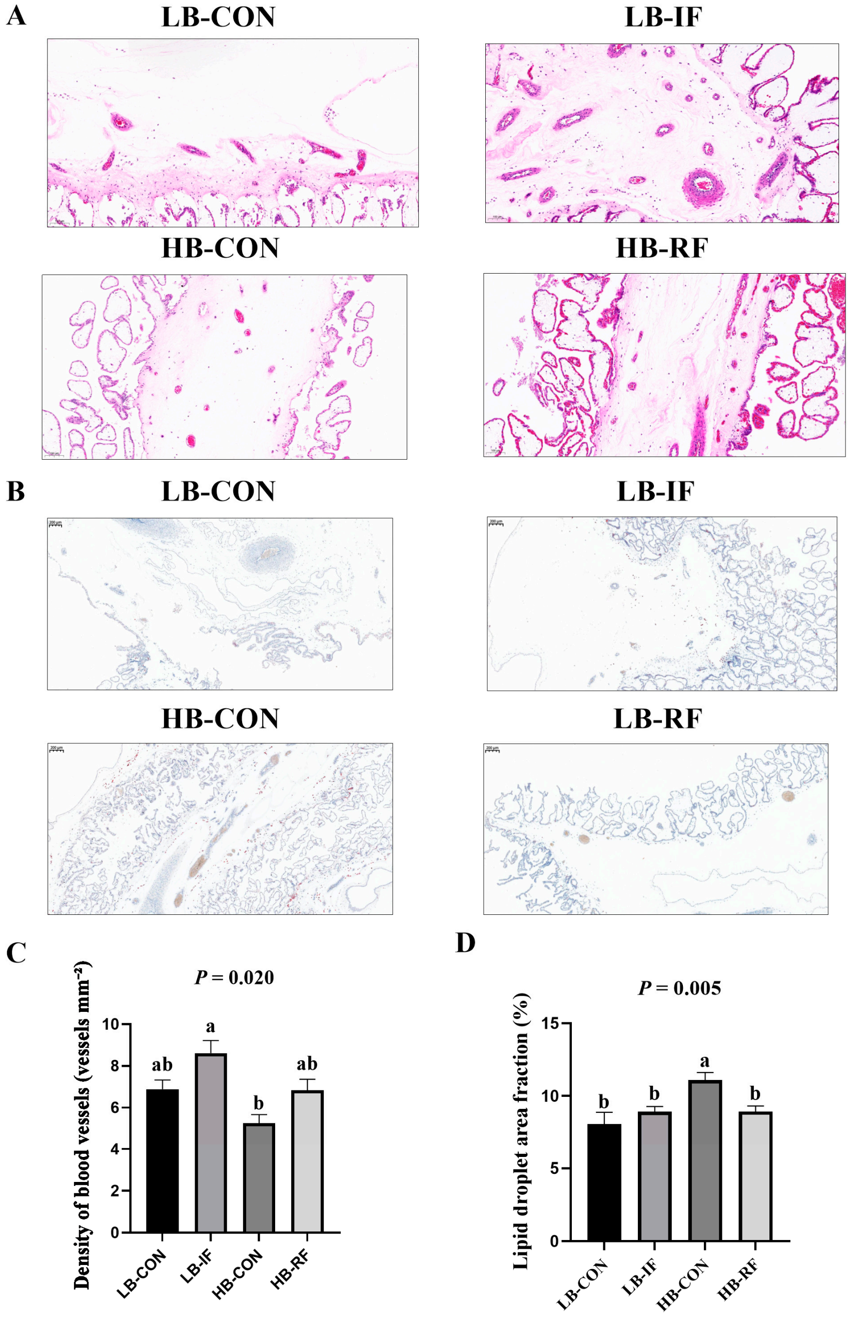

Supplementary Figure S1. Effects of REG on placental fat deposition and vessel density. (A) Hematoxylin and eosin staining of placenta tissues. (B) Oil Red O Staining of placenta tissues. (C) Quantification of the placental vessel density (n = 6). (D) Quantification of the placental fat deposition (n = 6). LB, backfat thickness of sows ranges from 12-16 mm. HB, backfat thickness of sows ranges from 18-22 mm. Sows in the LB-CON and HB-CON groups were fed a basic diet at a dosage of 1.88 kg/d. The LB-IF and HB-RF groups were fed a basic diet at dosages of 2.07 kg/d

and 1.69 kg/d, respectively. Values are presented as the means ± SEM. The different superscript letters on the same row indicate significant differences ($P < 0.05$).

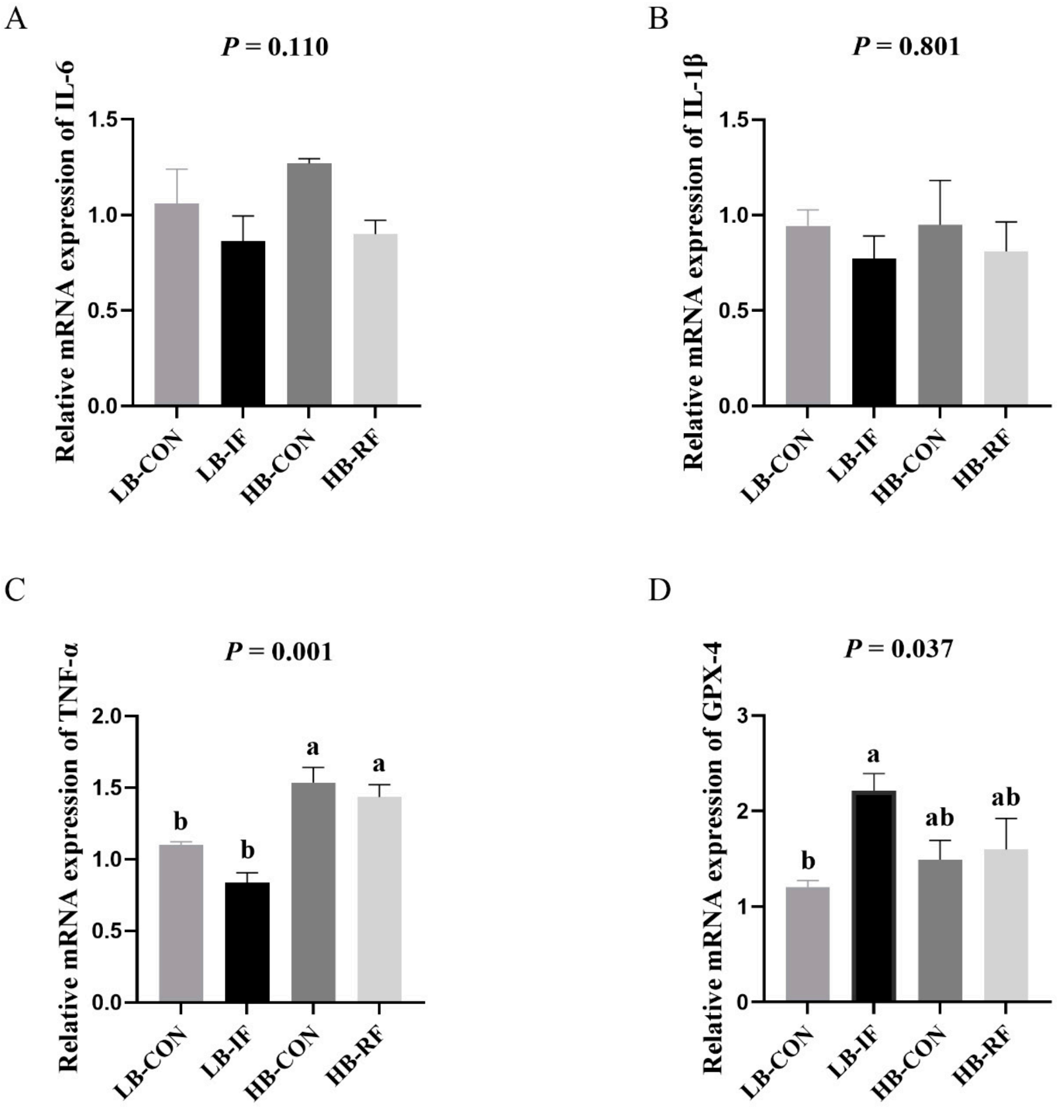

Supplementary Figure S2. Effects of REG on placental genes associated with antioxidant and inflammation in Shaziling sows. The relative mRNA expression levels of (A) interleukin-6 (IL-6), (B) interleukin-1β (IL-1β), (C) tumor necrosis factor-α (TNF-α), (D) glutathione peroxidase 4 (GPX-4).Values are presented as the means ± SEM. The different superscript letters on the same row indicate significant differences ($P < 0.05$).
